# A snapshot of translational research funded by the National Institutes of Health (NIH): A case study using behavioral and social science research awards and Clinical and Translational Science Awards funded publications

**DOI:** 10.1371/journal.pone.0196545

**Published:** 2018-05-09

**Authors:** Xueying Han, Sharon R. Williams, Brian L. Zuckerman

**Affiliations:** Science and Technology Policy Institute, Washington DC, United States of America; Institut Català de Paleoecologia Humana i Evolució Social (IPHES), SPAIN

## Abstract

The translation of biomedical research from basic knowledge to application has been a priority at the National Institute of Health (NIH) for many years. Tracking the progress of scientific research and knowledge through the translational process is difficult due to variation in the definition of translational research as well as the identification of benchmarks for the spread and application of biomedical research; quantitatively tracking this process is even more difficult. Using a simple and reproducible method to assess whether publications are translational, we examined NIH R01 behavioral and social science research (BSSR) awards funded between 2008 and 2014 to determine whether there are differences in the percent of translational research publications produced by basic and applied research awards. We also assessed the percent of translational research publications produced by the Clinical and Translational Science Awards (CTSA) program to evaluate whether targeted translational research awards result in increased translational research. We found that 3.9% of publications produced by basic research awards were translational; that the percent of translational research publications produced by applied research awards is approximately double that of basic research awards (7.4%); and that targeted translational research awards from the CTSA program produced the highest percentage of translational research publications (13.4%). In addition, we assessed differences in time to first publication, time to first citation, and publication quality by award type (basic vs. applied), and whether an award (or publication) is translational.

## Introduction

Translational research, in the public health and biomedical science context, was first defined by the Institute of Medicine’s Clinical Research Roundtable in 2000 as a two-phase process along the clinical research continuum moving from (1) basic science to clinical research (T1), and then (2) from clinical research to public health impact (T2) [1]. Many translational research frameworks have been espoused since then but most are a derivation of the original T1-T2 model [2–9]. For instance, the translational research framework by the National Center for Advancing Translational Sciences (NCATS) is a four-stage spectrum beginning with pre-clinical research (T1), followed by clinical research (T2), clinical implementation (T3), and public health (T4) [9]. According to NCATS, the translational research spectrum is better defined as an interconnected network where each stage can inform and learn from other stages. In this framework, basic research is not part of the translational research spectrum but is informed by other stages in the network. Despite numerous conceptualizations of the translational research continuum, there remains no clear consensus on which framework best conveys the process of translating basic science research to healthcare practices [10, 11]. It is commonly agreed on, however, that translational research bridges the gap ‘from bench to bedside’ and is both important and essential to improve public health. The push for increased translational research also stems from ever-present political pressure for the National Institutes of Health (NIH) to demonstrate that billions of tax-payer dollars are not wasted annually and that NIH-funded research produces tangible societal benefits [12–14].

In an effort to bridge the growing gap between basic and clinical research, former NIH Director Elias Zerhouni implemented the NIH Roadmap in 2003 [15]. The Roadmap (now named the Common Fund, https://commonfund.nih.gov/about/history) identified 28 large-scale, trans-institution initiatives under three overarching themes that the agency had prioritized for long-term research [16]. One of the themes was *Reengineering the Clinical Research Enterprise* in which the major focus was to facilitate the translation of basic research to clinical research [17]. As part of this effort, the National Center for Advancing Translational Sciences (NCATS) was established formally as one of the official NIH Institutes and Centers (IC) in 2012 with a mission to transform the translational science process. In addition, the Clinical and Translational Science Award (CTSA) program was established to “accelerate the translation of research discoveries from the bench to the bedside, train a new generation of clinical and translational researchers, and engage communities in clinical research efforts” (p. 41) [17]. CTSA is a national network that currently consists of 57 medical research institutions that work in conjunction with one another to facilitate the translational research process [18]. Translational research continues to be a priority for NIH and in fiscal year 2016, NIH allocated $500 million to the CTSA program [19].

As translational research gains momentum in both the biomedical and political sphere, it is important to develop methods to quantitatively assess the position of research on the translational pathway in order to determine the pace at which research is being translated from basic science to societal impacts, and the effectiveness of translational research targeted programs. A variety of methods to determine whether a study is translational have been suggested over the past few years. Weber (2013) proposed using a “triangle of biomedicine” to identify translational research [10]. The triangle consists of three topic areas, denoting the three corners of an equilateral triangle—animals and other complex organisms (A), cells and molecules (C), and humans (H). Individual articles are placed inside the triangle depending on how topically similar they are to each of the corners with those placed closer to H considered more translational. Additionally, publications can be grouped together by research area to estimate where a discipline falls along the translational research spectrum. Research areas that move closer to H over time indicate fields that are moving from bench to bedside.

Rosas et al. (2013) used citation data to evaluate the dispersion of scientific knowledge through time as well as the rate at which knowledge was spread by considering publication and citation dates [20]. The study modeled the time from publication of a clinical study to its citations, including citations in review articles, meta-analyses, and clinical guideline documents. Using 22 publications from the NIH HIV/AIDS Clinical Trials Networks, the authors found that half were cited by a clinical guideline within 12 months of publication, and all were included in a review within 24 months of publication.

Surkis et al. (2016) introduced a machine learning approach to identify and classify translational research [11]. The authors created a definitional checklist for each of the stages identified in the NCATS translational research spectrum and applied it to a subset of CTSA publications. The study identified basic biomedical research (T0) as studies that identify opportunities and approaches to health problems; translation to humans (T1) as studies that seek to move fundamental discovery into health applications, and/or provide clinical insights; translation to patients (T2) as health applications to implications for evidence-based practice guidelines; translation to practice (T3) as practice guidelines to health practices; and translation to communities (T4) as health practices to population health impacts, providing communities with optimal interventions (see reference [11] for full definitions and categorizations). The machine learning algorithm used a Bayesian logistic regression to process text from the title, abstract, and full MeSH terms/phrases associated with a publication to classify it along the translational research spectrum.

Schneider et al. (2017) presented a bibliometric approach to assess the research influence of 6 CTSA institutions [21]. Thomson Reuters and Elsevier performed bibliometric analyses and provided the authors with the results. Because it was a pilot study, both vendors provided the analyses at no cost. We realize, however, that contracting either of the two companies may pose a substantial cost to most researchers and that it may not be a feasible option to everyone. The metrics provided by Thomson Reuters and Elsevier were (1) number of publications, (2) average number of citations per publication, (3) percentage of publications in the top 10% per citations, and (4) comparative citation ratio. The authors found that publication output increased over time as CTSA institutions matured; that publications received more citations, on average, the longer they were published; and that CTSA publications were cited above the expected percentage rate. Unfortunately, the study did not identify what qualifies as translational research nor did it assess what bibliometric differences there are between translational and non-translational research.

Despite these studies, there remain a number of basic questions regarding translational research at the NIH level that have not been answered. For example, what percent of publications funded by NIH are translational? What percent of awards funded by NIH produce at least one translational publication? Do awards that focus on basic research produce less translational research than non-basic science awards? Does translational research get published or cited faster or slower than non-translational research? Our study is the first to answer these questions and more. Furthermore, we introduce a simple, straightforward method that can be easily replicated to categorize whether a publication should be considered translational research. This study takes a bibliometric approach and expands greatly on the work by Rosas et al. (2013) [20] and Schneider et al. (2017) [21] to assess the amount of translational research produced by basic research awards, applied research awards, and targeted translational research awards in addition to evaluating bibliometric differences between translational and non-translational research.

This study focuses specifically on behavioral and social science research (BSSR) related awards. We chose BSSR because of the simplicity in categorizing basic research awards and non-basic research awards—both *basic behavioral and social science* (bBSSR) and *behavioral and social science* are NIH research, condition, and disease categories (RCDC) spending categories that can be filtered through NIH RePORTER. This provided us with a simple way to differentiate basic and applied research within the same, larger research framework. According to the NIH Office of Behavioral and Social Sciences Research, studies that further the fundamental understanding of patterns in behavior or social functioning as related to health and well-being and their interactions with one another, biology, and the environment is considered bBSSR [22]. Conversely, research “designed to predict or influence health outcomes, risks, or protective factors; or is concerned with the impact of illness or risk for illness on behavioral or social functioning” is considered applied research (and is referred to “BSSR-only” within this study) [22]. In addition, it allowed us to test whether the triangle of biomedicine is an adequate tool to distinguish what is and is not translational research given that the subject matter in most behavioral and social science research is humans. We limited our analyses of BSSR related awards to R01 grants because they are the oldest and most common NIH award type.

In addition, to put BSSR awards into the larger context of NIH funded translational research, we evaluated 40,633 publications from 134 active and inactive CTSA centers and training grants (data provided by Surkis et al. 2016) [11]. Because CTSA is designed specifically to enhance translational research and BSSR awards do not have any intrinsic translational goals, this comparison provided us with the opportunity to assess if NIH’s translational efforts are indeed producing increased translational research. We hypothesized that CTSA awards should produce more translational research than BSSR awards.

## Methods

### Data collection

We limited our BSSR analyses to awards funded between 2008 and 2014. We selected these start and end dates because (1) 2008 was the first year in which the NIH Spending Category, a computerized process the NIH uses at the end of each fiscal year to classify research, condition, and disease categories (RCDC), was implemented (for more information on NIH spending categories, see reference [23]), and (2) to allow awards sufficient time to have published at least one publication. All active and inactive behavioral and social science research awards from 2008 to 2014 were downloaded from NIH RePORTER (https://projectreporter.nih.gov/reporter.cfm), an online repository of NIH-funded research projects. Basic behavioral and social science awards were defined as those that had a bBSSR RCDC tag and BSSR-only awards were defined as awards that were categorized by RCDC as BSSR but did not have a bBSSR tag (i.e., applied research awards).

We further limited these awards to those with an R01 activity code and those with application types of 1 (new application), 3 (supplements), or 5 (noncompeting continuations) (for more information about the different types of NIH applications, please see reference [24]). To guarantee that our final list of awards consisted of those that received its first year of grant support sometime between 2008 and 2014, we limited awards funded in 2008 to those with its first year of support; awards funded in 2009 to those with either its first or second year of support, and so on such that awards funded in 2014 were limited to those with its first, second,…, or seventh year of support. We removed awards that occurred less than the maximum number of years for which it received support. For example, an award with a project start date in 2002 but, for whatever reason, received its seventh year of support in 2014 would be removed from our dataset.

Next, all publications associated with the 6,387 awards were downloaded from NIH RePORTER. We note that only publications that cited grant support are retrievable from NIH RePORTER and included in our analyses. We make no observations regarding non-NIH funded research or NIH-funded publications that did not cite appropriate grant support. For each publication, we queried PubMed using the *rentrez* package [25] in R to retrieve the following information: the date that the article was submitted to the journal of publication, the date the article was accepted by the journal of publication, the electronic and in-print publication dates, the publication types of the article, all Medical Subject Headings (MeSH) descriptors associated with the article, and all of the associated grants cited by the article. We evaluated MeSH descriptors to assess whether the triangle of biomedicine would be able to distinguish translational vs. non-translational research in a research framework where the subject matter is human-centric. MeSH descriptors typically indicate the subject or topic area of an article, and were used by Weber (2013) as a way to identify translational research within the triangle of biomedicine [10]. Using the same protocol as Weber (2013), we classified publications that had one or more MeSH descriptors under the subtrees B01.050.150.900.649.801.400.112.400.400 (humans) and/or M01 (persons) as falling under the humans (H) topic area of the triangle of biomedicine. The proportion of bBSSR and BSSR-only publications with MeSH descriptors that would be classified as translational research under the triangle of biomedicine approach was calculated. A two-sample proportions test was used to evaluate whether the proportion of translational research publications differed between bBSSR and BSSR-only using the triangle of biomedicine approach.

For publications with both an electronic publication date and an in print publication date, the electronic publication date was chosen as it more accurately reflects when the scientific community was able to access a publication. In addition, because a lag between the electronic and in-print publication dates is common, the in print publication date may not reflect the full impact a publication has on its field in a timely manner. For publications in which only one type of publication date was provided, that date was used. We then limited publications to those that were published after 365 days from an award’s project start date to account for the lag-time between the receipt of an award and publishing results from that award. After data processing, a total of 1,341 bBSSR awards, 2,653 BSSR-only awards, and 29,894 distinct publications were used in our analyses.

#### Identifying primary and secondary translational research

We employed a similar approach used by Rosas et al. (2013) to classify whether a publication could be classified as translational research [20]. While Rosas et al. (2013) used data from PubMed to identify whether publications were meta-analyses, reviews, or guidelines, we used a much more comprehensive approach to classify publications. We identified all publication types that would report results from an original clinical study or trial, as defined by the 2017 list of Medical Subject Headings. Specifically, a publication was considered *primary translational research* (i.e., translational) if it was associated with at least one of the following publication types: clinical study; clinical trial; clinical trial, phase I; clinical trial, phase II; clinical trial, phase III; clinical trial, phase IV; controlled clinical trial; observational study; practice guideline; pragmatic clinical trial; or randomized controlled trial (for a complete list of publication types and their definitions, please see reference [26]).

In addition, we believe that studies that are not translational themselves may still contribute significantly to future translational research studies. We identified these as publications that did not fall into any of the eleven categories specified above but were cited by a publication that is considered translational. We have termed these as *secondary translational research*. To examine how many publications fall under the secondary translational research category and to estimate how fast knowledge is being passed through the translational research continuum, we chose a subset of bBSSR publications so that we could more clearly assess the length of time it takes for scientific knowledge to transition from basic science to the translational research spectrum. We limited the set of all bBSSR publications to those that were funded under a Request for Application (RFA) Funding Opportunity Announcement as these represented research areas that have been identified by one or more NIH institutes as crucial to accomplishing specific program objectives. A total of 1,393 distinct publications were identified using this criterion. Forward citation PubMed IDs (PMIDs) for the 1,393 publications were then manually retrieved from Scopus. Using the forward citation PMIDs, the following information was retrieved from PubMed for all forward citations: the date that the article was submitted to the journal of publication, the date the article was accepted by the journal of publication, the electronic and in-print publication dates, and the publication types of the article.

#### Citation analysis

We used the NIH iCite tool (https://icite.od.nih.gov/) to download bibliometric data for each of the 29,894 publications. In particular, iCite calculates a Relative Citation Ratio (RCR) metric for all NIH-funded publications. RCR uses each article’s co-citation network to field- and time-normalize the number of citations it has received [27]. Furthermore, RCR is benchmarked so that a publication that does not have any citations has a RCR score of 0 and that the mean paper receives a RCR score of 1.0. RCR values of less than 1.0 indicate publications that have had relatively little influence in their respective fields while values greater than 1.0 indicate those that have had more influence within their respective fields.

#### CTSA publications

Surkis et al. (2016) provided a list of PMIDs for 40,633 articles indexed to CTSA grants in PubMed [11]. We used this list of PMIDs to query PubMed and retrieve the publication type(s) for each article. As before, a publication was considered translational if it had at least one of the following publication types: clinical study; clinical trial; clinical trial, phase I; clinical trial, phase II; clinical trial, phase III; clinical trial, phase IV; controlled clinical trial; observational study; practice guideline; pragmatic clinical trial; or randomized controlled trial.

### Statistical analyses

#### Descriptive statistics

Less than 5% of publications (N = 1,033) cited both bBSSR and BSSR-only awards for funding support. These publications were included for both bBSSR and BSSR-only awards for all analyses. Summary statistics results may add up to more than 100% because of our decision to count these publications for both of their respective BSSR-related awards.

Generalized linear mixed-effects models with binomial distributions were used to assess the effects of *award type* (bBSSR vs BSSR) on whether publications were considered translational or not. The full model contained *award type* as a fixed effect and *grant number* as a random effect. A reduced mixed-effects model was generated containing only the random effect. A likelihood ratio test was then used to assess the significance of *award type* by comparing the reduced model to the full model.

For award-level analyses, an award was considered translational if it produced at least one translational publication. Generalized linear mixed-effects models with binomial distributions were run using the exact same fixed and random effects described above to determine if *award type* played a significant role in influencing whether an award produced at least one translational publication or not. A likelihood ratio test was used again to assess the significance of *award type* by comparing the reduced model to the full model. All generalized linear mixed-effects models were generated using the glmer function in the lme4 package in R [28].

#### Time to first publication

The time to first publication analysis was limited to publications that provided, minimally, the month and year of an article’s publication date. Time to first publication was calculated as the number of days between an award’s project start date and the publication date for the first publication recorded for that award. All subsequent publications for an award were excluded from this analysis. For publications in which only the month and year were provided, the *day* unit was set to the first of the month. Awards that had zero publications as of 7 August 2017, the date on which publication data were retrieved from NIH RePORTER, were right censored. A Cox proportional hazard model was used to examine whether *award type* (bBSSR vs BSSR), *whether a publication was translational or not*, and *number of acknowledged grants* were predictive of time to first publication. A type-III analysis of deviance had shown that none of the two-way, three-way, or four-way interactions of the variables were significant at the α = 0.05 level and therefore no interactions were included in the final Cox regression model.

#### Time to first citation

The time to first citation analysis was limited to the 1,393 bBSSR, RFA publications that were identified using our selection criteria. Publications with only “year” as the publication date were excluded from this analysis (N = 90). Forward citation PMIDs were unavailable for 374 publications and were, therefore, right-censored. Time to first citation for censored publications was calculated as the number of days between an article’s publication date and the date when forward citation data were downloaded from Scopus (31 August 2017). For publications with citations, time to citation was calculated as the number of days between an article’s publication date and the publication date of that article’s first forward citation. All subsequent forward citations of an article were excluded from this analysis.

In cases in which the time to first citation was negative (i.e., a publication was cited before it was officially published), the value for time to first citation was set to zero. Cox proportional hazard models were used to assess if the time to first citation differed significantly by whether a publication was a secondary translational research publication or not.

#### Citation analysis

We used a multiple rank-based regression—a non-parametric, robust alternative to traditional likelihood or least squares estimators [29]—to determine the effects of *award type*, and *whether a publication was translational or not* on Relative Citation Ratio (RCR). The full rank-based regression model consisted of both explanatory variables whereas the two reduced models consisted of each individual explanatory variable. Rank-based regression models were generated using Wilcoxon regression rank scores calculated by the rfit function in the Rfit package in R [29]. To determine whether each explanatory variable was significant in explaining the variation in RCR values, the drop.test function in the Rfit package was used to calculate the reduction in dispersion between each of the reduced models and the full model.

#### In comparison with CTSA publications

Pairwise comparison of proportions was used to assess whether the proportion of translational publications differed by award type (in this case, bBSSR vs BSSR vs CTSA).

**Fig 1:**
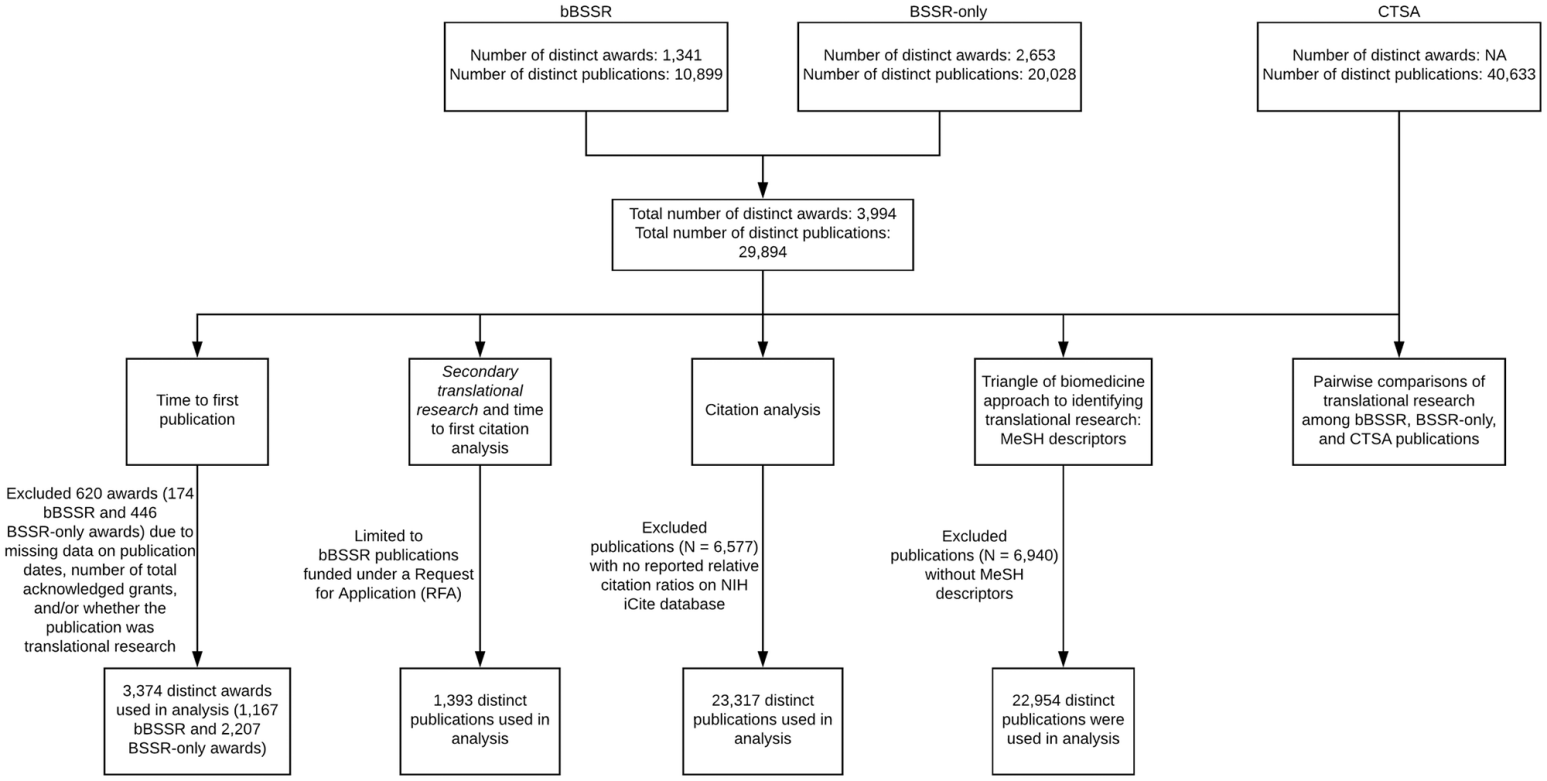
Flow diagram of awards and/or publications that are included (and excluded) for each analysis.

For ease, we have provided a flow diagram displaying what was included (and excluded) for each analysis (Fig 1). All analyses were performed in R. All data (raw and derived) and associated R codes can be found at Figshare (https://doi.org/10.6084/m9.figshare.c.3908134).

## Results

### Descriptive statistics

**Fig 2:**
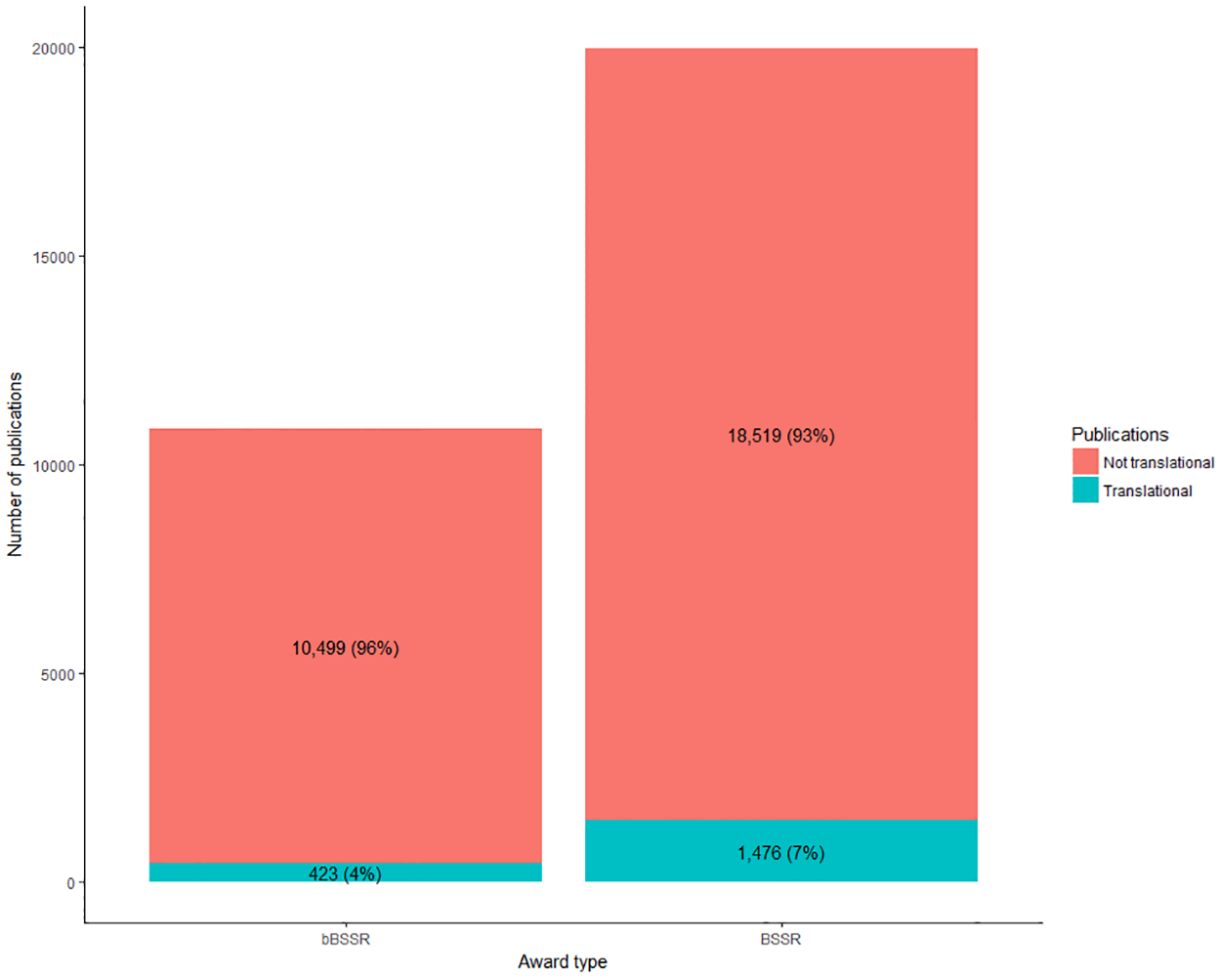
Total number of publications by award type (bBSSR or BSSR-only) and whether a publication was considered primary translational research.

Of the 29,894 distinct publications used in our analyses, 10,899 (36%) were funded by bBSSR awards, and 20,028 (67%) were funded by BSSR-only awards (Fig 2). A total of 1,826 distinct publications (6.1% of all publications) were translational, 423 (23%) of which were funded by bBSSR awards, and 1,476 (81%) of which were funded by BSSR-only awards.

At the publication level, results from the generalized linear mixed-effects models showed that *award type* (Χ^2^_1_ = 120, P < 0.001) was a significant factor in determining whether a publication was translational or not. Specifically, the percent of translational bBSSR publications (3.9%) was significantly lower than that of translational BSSR-only publications (7.4%). At the award level, *award type* was also a significant factor (Χ^2^_1_ = 101, P < 0.001) in determining whether an award produced at least one translational publication. Similar to the publication level analysis, the percent of bBSSR awards that produced at least one translational publication (15%) was significantly lower than that of BSSR-only awards (28%).

Of the 22,954 publications for which MeSH descriptors were retrieved, 18,839 (82%) had a “humans” and/or a M01 (persons) MeSH descriptor. By award type, 73% (6,018 out of 8,256 publications) of bBSSR and 87% (13,548 out of 15,548 publications) of BSSR-only publications, had a “humans” and/or a M01 (persons) MeSH descriptor. A two-sample proportion test showed that using the triangle of biomedicine approach, the proportion of BSSR-only publications that had a “humans” and/or a M01 (persons) MeSH descriptor was significantly higher than the proportion of bBSSR publications.

### Time to first publication

**Fig 3:**
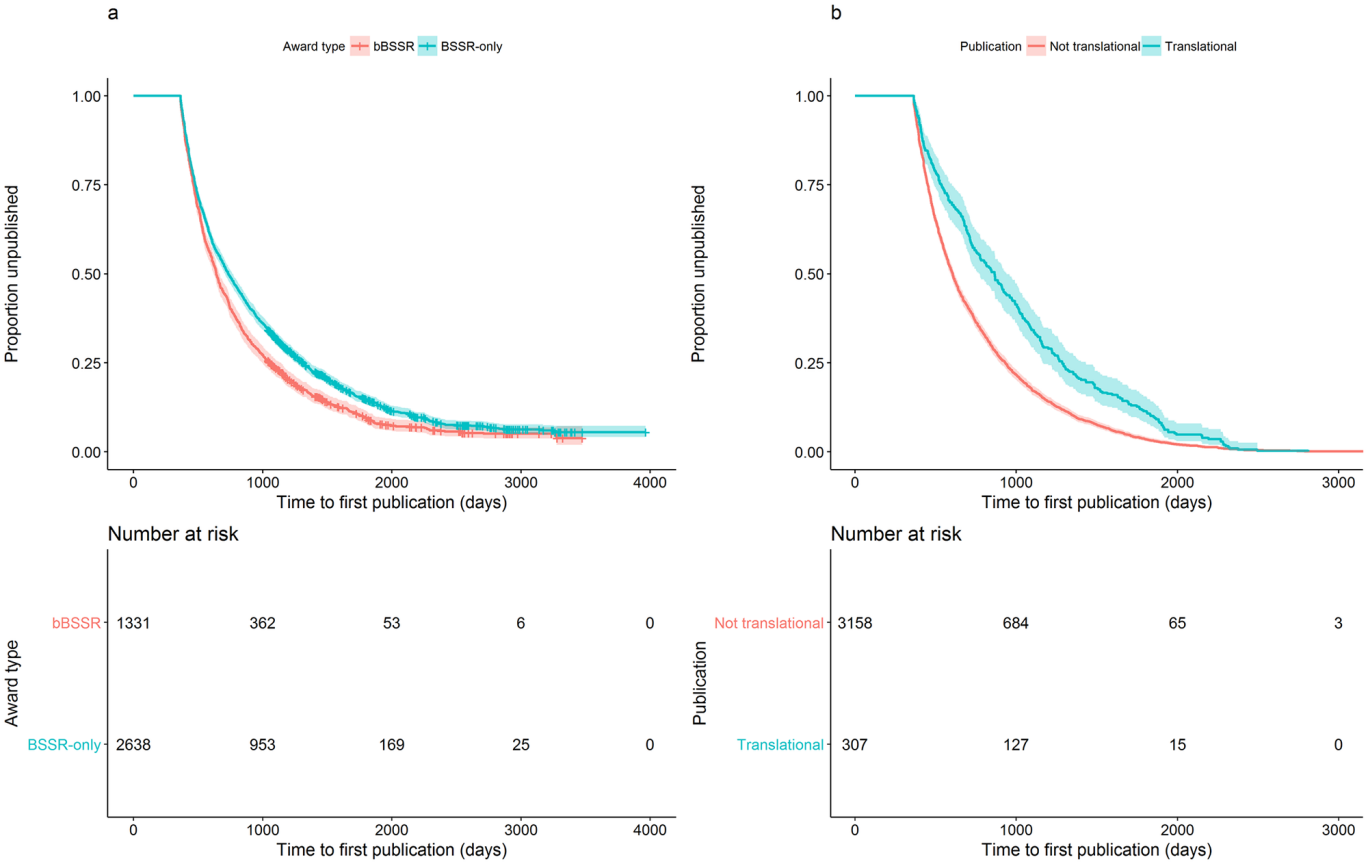
Proportion of awards not published by (a) award type (bBSSR or BSSR-only) and (b) whether a publication was considered a translational research publication. The hazard rate to first publication was significantly slower for BSSR-only compared to bBSSR awards (0.86, P < 0.001), and for translational compared to non-translational awards (0.68, P < 0.001).

**Fig 4:**
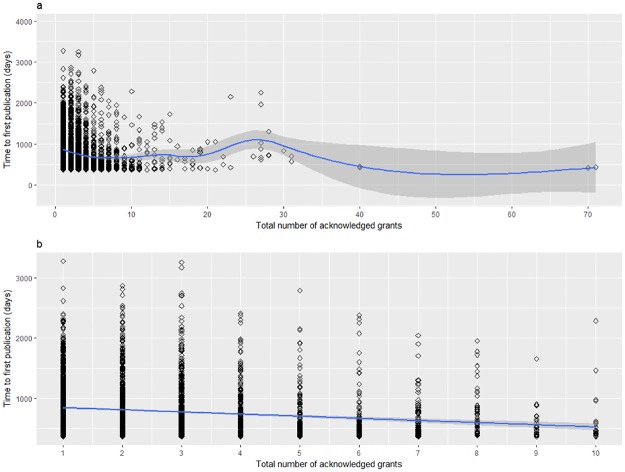
Time to first publication (days) by total number of acknowledged grants for (a) all grants, and (b) up to the first 10 grants acknowledged in bBSSR and BSSR-only publications.

Results from the time to first publication Cox regression model showed that all three factors (*award type*, *whether a publication was translational or not*, and *number of acknowledged grants*) significantly influenced the rate to first publication. Specifically, the rate to first publication was significantly slower for BSSR-only compared to bBSSR awards, and for translational compared to non-translational awards (Fig 3, Table 1). For number of acknowledged grants, the hazard ratio for each additional grant is 1.02 (1.01 to 1.03) indicating that the rate to first publication is 1.02 times faster for each supporting grant within a study. In general, the rate of first publication between an article with *m* funding sources compared to one with *n* funding sources, where *m* > *n*, will be 1.02^*m-n*^ times faster. For example, the rate of first publication for an article that acknowledged 10 different funding mechanisms compared to one with one funding source would therefore be (1.02)^10-1^ = 1.20 times faster. A more in-depth look at the effects of multiple funding sources on time to first publication revealed that the decrease in time appeared to be limited and was strongest within the first ten grants (Fig 4). A strong, linear negative relationship between time to first publication and number of acknowledged grants can be observed for the first ten grants but beyond this point, the relationship turns positive before becoming negative again. Furthermore, the 95% confidence interval increases in size and we cannot be certain of the relationship between number of grants and time to first publication given the small number of observations.

**Table 1:** Time to first publication Cox proportional hazard model results.

Factors	Hazard ratio (95% CI)	Mean (± 1 SE) time (days) to first publication	P-value
Award type:			<0.001
bBSSR	1.00	830 (± 14.4)	
BSSR-only	0.86 (0.80 to 0.92)	941 (± 11.8)	
Whether a publication is translational or not:			<0.001
Not translational	1.00	762 (± 7.7)	
Translational	0.68 (0.61 to 0.77)	987 (± 30.6)	
Number of acknowledged grants	1.02 (1.01 to 1.03)		<0.001

### Time to first citation

Recall that we defined *primary translational research* (referred simply as “translational”) as any article with one of the eleven publication types identified previously, and *secondary translational research* as any publication that is not translational but is cited by a translational publication. Of the 1,393 distinct publications for which we attempted to retrieve forward citation PMIDs, 58 were primary translational research (4.3%) and 192 were secondary translational research (16%). Of the 11,604 distinct forward citation PMIDs that were retrieved, 471 (4.1%) were translational.

**Fig 5:**
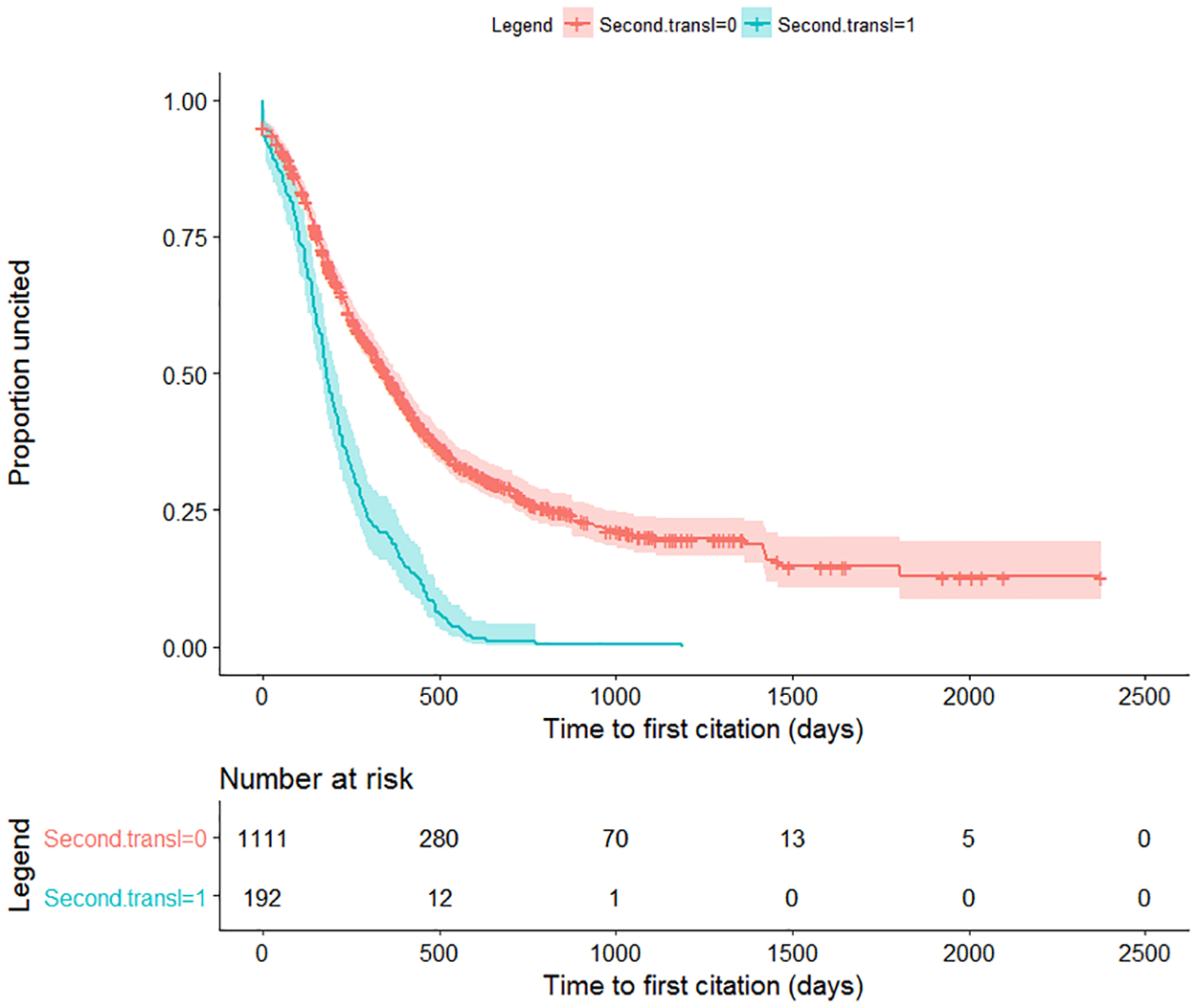
Proportion of publications not cited according to whether a publication was considered a secondary translational research publication. The hazard for secondary translational research publications compared to publications without translational forward citations was 2.4 (95% confidence interval of 2.1 to 2.8, P < 0.001).

Results from the Cox regression model showed that the rate of first citation differed significantly depending whether a publication was a secondary translational research publication or not. The hazard for secondary translational research publications compared to publications without translational forward citations was 2.4 (95% confidence interval of 2.1 to 2.8, P < 0.001) indicating that the rate of first citation for secondary translational research publications was approximately two and a half times faster than those that have not been cited by translational publications (Fig 5). The mean (± 1SE) time to first citation for secondary translational research publications was 219 (± 12) days and 376 (± 10) days for publications without a translational forward citation.

### Citation analysis

Results from the multiple rank-based regression indicated that both *award type* (F_1, 23,314_ = 18.1, P < 0.001) and *whether a publication was translational or not* were significant (F_1, 23,314_ = 49.9, P < 0.001). Specifically, BSSR-only publications had significantly higher RCRs (median: 1.48) than bBSSR publications (1.40); and translational publications had significantly higher RCRs (1.74) than non-translational publications (1.43). The range of RCR values varied widely for both bBSSR (0 to 206) and BSSR-only publications (0 to 145).

### CTSA publications

Of the 40,633 distinct CTSA funded publications identified by Surkis et al. (2016), 5,451 (13.4%) were translational. Results from the pairwise comparison of proportions for bBSSR, BSSR-only, and CTSA publications showed that bBSSR publications had a significantly lower percent of translational publications (3.9%) than both BSSR-only and CTSA (P < 0.001 for both); the percent of BSSR-only publications (7.4%) was significantly higher than that of bBSSR (P < 0.001) but significantly lower than that of CTSA publications (13.4%) (P < 0.001); and CTSA publications had the highest percent of translational publications (P < 0.001 for comparing to both percent of translational research bBSSR and BSSR-only publications).

## Discussion

### Limitations to the study

We acknowledge that this study focuses only on NIH grants awarded within the behavioral and social science research field and that our results may not be applicable to all research areas. We encourage future studies to evaluate the level of translational research produced in other research fields supported by the NIH. In addition, we recognize that the PubMed publication type may not be one-hundred percent accurate in reflecting the true nature of the actual research done. We note, however, that the U.S. National Library of Medicine (NLM) has been indexing publication types by trained MEDLINE indexers since 1991 and no publication type is assigned by a computer algorithm (authors’ personal communication with NLM customer service). As such, we believe that the 11 publication types that are used in this study to classify translational research is a suitable and reliable form of categorizing whether a publication is translational research. Lastly, we acknowledge that the list of publications from Surkis et al. (2016) is unlikely to be a perfect representation of the publications that have emanated from NCATS-supported research as some authors do not provide funding information when publishing. The list of CTSA-related publications is, therefore, likely an underrepresentation of the total number of CTSA-funded publications. Furthermore, because as many as 50% of clinical trials go unpublished [30], it is quite possible, as well as probable, that there are CTSA funded studies that resulted in translational research but are simply not published in scientific journals. Our finding that 13.4% of CTSA-related publications are primary translational research is likely an under-representation of the true level of translational research actually taking place by CTSA studies.

### Translational research funded by the NIH

This study provides the first estimates of translational research outputs produced by NIH funded studies within the behavioral and social science field. At the publication level, we found that 3.9% of publications funded by basic research awards (i.e., bBSSR), 7.4% of publications funded by applied research awards (i.e., BSSR-only), and 13.4% of CTSA, a targeted translational research program, publications could be considered primary translational research. At the award level, the numbers are a little higher, we found that 15% of bBSSR awards and 28% of BSSR-only awards produced at least one primary translational research publication. Unfortunately, we were unable to calculate the percent of CTSA awards that produced at least one translational publication as only the PMIDs were made publically available. Interestingly, qualifying whether publications are translational research using the triangle of biomedicine approach, while vastly overestimating the level of translational research for bBSSR and BSSR-only publications compared to our approach, also indicated that a significantly higher proportion of BSSR-only publications could be considered translational research than bBSSR publications. Most importantly, these findings show that for NIH-funded behavioral and social sciences research, there is a natural progression of increased translational research as we move across the research continuum from basic science to applied science. Furthermore, we found that the level of translational research was highest under a targeted translational research program. This suggests that (1) translational research can happen at any stage of the research continuum, albeit at a lower rate closer to the basic science side, and (2) targeted translational research programs such as CTSA appear to be effective at promoting the translation of basic and applied sciences to healthcare outcomes and impacts.

Results with regard to time to first publication were as expected. As bBSSR awards are likely to be more focused on lab sciences as opposed to clinical sciences like BSSR-only awards, and therefore, may not involve institutional review board (IRB) approvals, it would be logical for bBSSR awards to have a faster rate of first publication. It is also reasonable for awards that published a translational publication as their first publication to take longer than those that published a non-translational publication since clinical studies can take a long time to receive human subjects approval, find eligible participants for the study, and for the study to actually take place. Lastly, our finding that time to first publication decreases by 2% for each additional source of funding support (up to the first 10 sources) is consistent with the idea that many studies are not standalone studies anymore, and that many use previous (or currently) funded studies as jumping off points, which would accelerate time to first publication.

With regard to citation analysis, RCR provided a feasible way to assess citations normalized by year and field. We found that, on average, bBSSR and BSSR-only publications had RCR values above 1.0 indicating they had relatively more influence compared to the average paper within their respective fields. Overall, BSSR-only publications had relatively higher research influence than bBSSR publications, and translational publications had relatively higher research influence than non-translational publications.

### Secondary translational research

We believe that in addition to primary translational research, it is also important to consider secondary translational research. Studies that are not translational themselves but that contribute directly to translational research shows movement along the research continuum. We found that among secondary translational research publications, it takes, on average, approximately 2.3 years for advances from basic scientific research to get incorporated into the translational research spectrum. The vast majority (97%) of the translational forward publications were some form of clinical trial; however, 12 were classified as practice guidelines. These 12 publications cited 10 of the non-translational bBSSR publications (0.1%) with time to citation ranging from 177 to 1,585 days (or approximately 0.5 to 4.3 years). This shows that, although rare, scientific knowledge derived from basic scientific research can be integrated into healthcare practices in a relatively short amount of time.

Within the smaller subset of bBSSR publications for which we manually retrieved forward citations, we found that the time to first citation for secondary translational research publications was approximately 2.5 times faster than publications that did not get cited by a translational publication. A possible explanation for this observed difference is that secondary translational research publications may have made an important scientific discovery or contribution that was important to moving research in its particular field down the research continuum. This could be why secondary translational research publications received their first citation at a much faster rate and why they were cited by at least one translational publication. Previous studies have shown that clinical research studies that produced results of high scientific importance were significantly more likely to both publish [31] and publish at a significantly faster rate (about 2.4 times faster) than studies with results that are of low scientific importance [32]. In addition, studies that found significant results published at a faster rate than those that found null results, and studies that found positive significant results published faster than those that found negative significant results [32–35]. It is likely that not only do studies with high scientific importance get published at a much faster rate but are also cited faster. We acknowledge that we did not assess whether the first forward citation was a self-citation but we believe that given self-citations would be present in both secondary translational research publications and those that are not, and given that the magnitude of difference observed the time to first citation between secondary translational research publications and those that are not, we believe the difference in time to first citation between the two groups would still be significant.

### Identifying translational research

In this study, we considered all research that facilitates the translation of basic and applied research findings into healthcare practices and outcomes as translational research. Because qualifying whether studies are translational research is incredibly difficult, we chose to use publication types as a proxy for whether a study could be considered translational research. We have shown in this study a simple, reproducible, and comprehensive method to assess whether a publication is translational or not. Our method utilizes publically available information and does not require any substantial monetary resources aside from a researcher’s time. Although classification along the translational research spectrum (e.g., T1, T2, etc.) was not the goal of this study, our protocol of identifying translational research can be easily expanded to do so. For example, Surkis et al. (2016) specified that Phase 1 clinical trials fall under the T1 phase, that Phase 2 and Phase 3 clinical trials fall under T2, that Phase 4 clinical trials fall under T3, and that publications and documents that focus on broader implementation of improved practices or interventions, such as practice guidelines, would fall under the T4 phase [11]. Given that practice guidelines and different phases of clinical trials are identified as individual publication types for articles indexed in PubMed, it would be easy to assign articles to different phases along the translational research spectrum based on an article’s publication type.

We further showed that depending on the type of research, particularly those that focus on human subjects such as BSSR research, the triangle of biomedicine may not be able to distinguish non-translational from translational research through the use of MeSH descriptors alone. Approximately four-fifths of BSSR related publications (73% of bBSSR and 87% of BSSR-only publications) had a “humans” and/or a M01 (persons) MeSH descriptor and would have been considered translational by the triangle of biomedicine while in reality, only 3.9%% of bBSSR and 7.4% of BSSR-only publications were translational (as defined in this study). For studies that focus on human subjects, the triangle of biomedicine would vastly overestimate the number of translational research publications compared to our approach.
